# Molecular docking analysis of phytoconstituents from *Nilavaagai Chooranam* with histamine H1 receptor

**DOI:** 10.6026/973206300213511

**Published:** 2025-10-31

**Authors:** Vinu Bharathi Balasubramaniam, Vinodini Ramamoorthy, Saraswathi Balasubramanian, Siva Annamalai, Deepa Ravichandran

**Affiliations:** 1 Department of Clinical Research, Siddha Regional Research Institute, Central Council Research in Siddha, Ministry of Ayush, Government of India, Kuyavarpalayam, Puducherry, India; 2 Department of Clinical Research, Central Council Research in Siddha, Ministry of Ayush, Government of India, Tambaram Sanatorium , Tamil Nadu, Chennai, India; 3 Assisstant Medical Officer (Siddha), Government Hospital, (Under directorate of Indian medicine and Homeopathy), Kilvelur, Nagapattinam, Tamil Nadu, India; 4 Siddha physician, Om Muruga Siddha clinic, Thanjavur, Tamil Nadu, India

**Keywords:** *Nilavaagai Chooranam*, Histamine H1 receptor, cetirizine, *in silico* analysis, molecular docking study, siddha

## Abstract

*Nilavaagai Chooranam*, a polyherbal formulation in Siddha medicine, is traditionally used for treating respiratory and allergic conditions. Histamine H1 receptors are central to allergic reactions and modulating their activity can help manage conditions like allergic rhinitis and asthma. The objective of this study is to investigate the potential of bioactive compounds in *Nilavaagai Chooranam* to modulate histamine-mediated allergic responses by targeting the histamine H1 receptor. Molecular docking analysis was conducted to evaluate the binding affinities of key phytochemicals Kaempferol, Gingerenone-A, Piperic acid, Embelin, Carvone, and β-pinene against the H1 receptor (PDB ID: 3RZE). Cetirizine, a well-known H1 antagonist, was used as a reference ligand. Significant interactions were observed between the phytochemicals and the H1 receptor. Gingerenone-A and Embelin exhibited the highest binding affinities, with interactions involving key residues like Trp428, Tyr431, and Phe432. These results were comparable to those of Cetirizine. Data shows that *Nilavaagai Chooranam* contains compounds with anti-allergic and anti-inflammatory potential, supporting its traditional use in treating histamine-mediated conditions. Further *in vitro* and *in vivo* studies are needed to validate these *in silico* results and explore the formulation's potential as a natural alternative to synthetic antihistamines.

## Background:

Nilavakai curanam (NC), a Siddha polyherbal formulation, is traditionally used to treat a range of health conditions such as indigestion, bloating, vomiting, cough and bronchial asthma [1]. The formulation's effectiveness is attributed to its constituent herbs, which possess bronchodilatory, antimicrobial, anti-inflammatory, immunomodulatory, antihistamine properties, insecticidal, anti-asthmatic, anti-inflammatory, anti-cancer, antiproliferative activity [2]. Histamine H1-receptors play a significant role in the pathological processes associated with allergies. Clinical trials have shown that H1-receptor antagonists are effective in alleviating symptoms such as sneezing, itching and rhinorrhea, which are characteristic of allergic rhinitis [3]. In the lungs, H1-receptors mediate the bronchoconstrictive effects of histamine and contribute to increased vascular permeability, leading to plasma exudation [4]. These receptors are also expressed on T cells, B cells, monocytes and lymphocytes, where their activation exerts pro-inflammatory effects [5]. It has been suggested that H1-receptor signaling may enhance antigen receptor-mediated signaling pathways, promoting lymphocyte proliferation and stimulating cytokine and antibody production by T and B cells, respectively [6]. This indicates that H1-receptors have a broader immunomodulatory role in inflammatory processes beyond their classical actions on smooth muscle and endothelium. Given that the ingredients of NC have documented antihistamine and anti-inflammatory activities, it is plausible that they exert their therapeutic effects by modulating the activity of H1-receptors [7]. Therefore, it is of interest to document the molecular docking analysis of the phytoconstituents present in Nilavakai curanam with the histamine H1 receptor.

## Materials and Methods:

## Phytocomponent Retrieval and target protein:

The active compounds, or phytochemicals, within the formulation NC were identified through a thorough review of scientific literature (Table 1 - see PDF). These phytochemicals are believed to interact with biological targets to exert their effects. The histamine H1 receptor, a key target in the management of allergic conditions, was chosen for this study. The binding interactions between the phytochemicals and this receptor were analyzed to assess their potential as therapeutic agents. Cetirizine, a well-established antihistamine drug, was included as a standard reference compound to compare the binding affinity and interaction profiles of the identified phytochemicals. The 2D and 3D structures, docking pose with the Histamine H1 receptor (PDB ID: 3RZE), and 2D interaction plot analysis of the standard drug cetirizine are presented in Figure 1 (see PDF).

## Target protein preparation:

The core amino acid residue, tryptophan 428 (Trp428), of the histamine H1 receptor is crucial for binding substrates and inhibitors [13]. By forming hydrogen bonds with these residues, phytocomponents can potentially inhibit the function of the H1 receptor. This inhibition may offer therapeutic benefits for managing allergic conditions. To explore this potential, the 3D structure of the histamine H1 receptor (PDB ID: 3RZE) was retrieved from the Protein Data Bank (Figure 2 - see PDF). The protein preparation involved a comprehensive clean-up process, including the addition of essential missing hydrogen atoms. Using the AutoDock program, various orientations of the phytocomponent molecules relative to the target protein were evaluated. The most favorable docking poses were identified based on detailed interaction analyses, highlighting the binding affinity and the potential inhibitory effects of the phytocomponents on the H1 receptor [14].

## Ligand preparation:

Each selected phytochemical was prepared for molecular docking by retrieving its 2D and 3D structures from chemical databases or generating them using molecular modeling software. Energy minimization was then performed to achieve stable conformations and reduce steric hindrance. Subsequently, each ligand was parameterized by assigning appropriate partial charges and defining rotatable bonds to allow for flexible interaction with the target protein. The structures of the ligands are presented in Figure 3 (see PDF) and their physicochemical properties are summarized in Table 2 (see PDF) [15].

## Docking procedure:

Docking calculations were carried out for retrieved phytocomponents against target enzyme H1 receptor. Essential hydrogen atoms, Kollman united atom type charges, and solvation parameters were added with the aid of AutoDock tools Affinity (grid) maps of xx Å grid points and 0.375 Å spacing were generated using the Autogrid program. AutoDock parameter set- and distance-dependent dielectric functions were used in the calculation of the van der Waals and the electrostatic terms, respectively. Docking simulations were performed using the Lamarckian genetic algorithm (LGA) and the Solis & Wets local search method. Initial position, orientation, and torsions of the ligand molecules were set randomly. All rotatable torsions were released during docking. Each docking experiment was derived from 2 different runs that were set to terminate after a maximum of 250000 energy evaluations. The population size was set to 150. During the search, a translational step of 0.2 Å, and quaternion and torsion steps of 5 were applied [16, 17].

## Results and Discussion:

The binding interactions of selected phytochemicals from NC, along with the standard antihistamine drug cetirizine, were analyzed against the Histamine H1 receptor. The number of hydrogen bond interactions and the key amino acid residues involved in the ligand-receptor binding were identified. Among the tested compounds, Gingerenone-A exhibited the highest number of interactions, forming hydrogen bonds and hydrophobic contacts with several active site residues including ASN84, ASP107, TYR108, SER111, and TYR458. Kaempferol, Embelin, and Piperic acid also showed notable binding, interacting with key residues such as TYR108, SER111, ASN198, and PHE432. Carvone and β-Pinene showed relatively fewer interactions. The standard drug cetirizine demonstrated strong binding affinity with the receptor, interacting with critical residues such as ASP107, TYR108, SER111, THR112, and TYR458, which are known to be important for H1 receptor antagonism. The presence of interactions with residues common to both cetirizine and the phytochemicals suggests that these natural compounds may mimic the binding behaviour of standard antihistamines. The summary of the molecular docking studies of Phytochemicals against histamine H1 receptor (PDB) - 3RZE are tabulated in Table 3 (see PDF) and Amino acid Residue Interaction of Lead against Histamine H1 receptor (PDB) - 3RZE are outlined in Table 4 (see PDF). Likewise the Docking poses of phytochemicals with Histamine H1 receptor and 2D interaction plot analysis of Phytochemicals are depicted in Figure 4 (see PDF) and 5 (see PDF).

In this study, the molecular docking analysis demonstrated that multiple bioactive compounds present in NC exhibited strong interactions with the histamine H1 receptor, suggesting their potential role in modulating histamine-mediated allergic responses. The compounds Kaempferol, Gingerenone-A, Piperic acid, Embelin, Carvone, and β-pinene, known for their varied pharmacological properties, were analyzed, along with Cetirizine, a well-established H1-receptor antagonist, as a control [18]. The docking results revealed significant binding affinities of these phytocomponents to the histamine H1 receptor. Kaempferol, Gingerenone-A, Piperic acid, Embelin, and Carvone exhibited binding affinities ranging from -6.52 kcal/mol to -9.12 kcal/mol, indicating promising interactions at the receptor's active site. In particular, Gingerenone-A, with a binding energy of -9.12 kcal/mol and an inhibition constant (Ki) of 205.32 nM, demonstrated the strongest affinity among the tested phytocomponents. This was closely followed by Embelin, with a binding energy of -8.80 kcal/mol and Ki of 355.26 nM. The relatively high binding affinity of these phytocompounds can be attributed to their ability to form hydrogen bonds and other stabilizing interactions with key residues of the histamine H1 receptor, such as Trp428, Tyr431, and Phe432 [19]. Comparatively, Cetirizine, used as a reference ligand, exhibited the highest binding energy (-11.38 kcal/mol) and the lowest inhibition constant (4.52 nM), reflecting its potent antihistamine effect [20]. However, the phytocomponents tested in this study also demonstrated comparable binding interactions, indicating that these natural compounds might offer alternative therapeutic potential in managing allergic responses, particularly for those seeking plant-based remedies. Hydrogen bond analysis showed that the interaction between phytocomponents and critical residues within the receptor's binding pocket plays a pivotal role in stabilizing the ligand-receptor complex [21]. The hydrogen bonding with Trp428, a core amino acid residue, was observed in most of the docked compounds, highlighting its importance in ligand binding. For instance, Kaempferol and Gingerenone-A formed hydrogen bonds with both Tyr107 and Asp108, key residues that contribute to stabilizing the ligand in the binding site, thereby enhancing receptor inhibition. Hydrophobic interactions also contributed to the overall binding affinity, particularly with residues such as Tyr112 and Phe435. The docking results suggest that NC contains compounds capable of modulating the histamine H1 receptor, thereby offering anti-allergic and anti-inflammatory effects. Given the role of histamine H1 receptors in allergic rhinitis, asthma, and other inflammatory conditions, these findings provide a molecular basis for the traditional use of NC in Siddha medicine to treat respiratory and allergic disorders [22]. Phytochemicals like Kaempferol and Gingerenone-A not only exhibit strong receptor interactions but also possesses documented anti-inflammatory, antioxidant, and immunomodulatory properties, which may further contribute to their therapeutic efficacy. Furthermore, the diversity of interactions observed between the different phytochemicals and the histamine H1 receptor suggests that NC may act through a synergistic mechanism, where multiple compounds work in concert to achieve the observed therapeutic effects. This hypothesis is consistent with the principles of polyherbal formulations in traditional medicine, where the combination of different herbs is believed to enhance efficacy through complementary actions. When compared to conventional antihistamines like Cetirizine, the phytocomponents of NC offer a natural alternative with potentially fewer side effects. While Cetirizine demonstrated superior binding in this study, the phytocompounds, particularly Gingerenone-A and Embelin, still exhibited strong receptor interactions that could translate to meaningful biological effects. Importantly, the lower molecular weight and natural origin of these compounds may offer an advantage in terms of bioavailability and safety, making them attractive candidates for further *in vivo* studies [23]. Despite the promising *in silico* results, there are limitations to this study. Molecular docking provides insights into binding affinity and potential interactions, but it does not account for the pharmacokinetics, bioavailability, or metabolism of these compounds in the human body. Future studies should focus on validating these findings through in vitro and *in vivo* experiments to assess the actual biological activity of these phytocompounds. Additionally, exploring the synergistic effects of combining multiple phytocomponents could further clarify the therapeutic potential of NC. Therefore, integrating docking studies with dynamic simulations or exploring other receptor subtypes involved in allergic responses could provide a more comprehensive understanding of the formulation's effects.

## Conclusion:

Several phytochemicals in NC, including Kaempferol, Gingerenone-A, Piperic acid, Embelin, and Carvone, exhibit promising interactions with the histamine H1 receptor, offering potential anti-allergic and anti-inflammatory benefits. While further experimental validation is needed, the results support the traditional use of NC in treating histamine-mediated allergic conditions and open avenues for the development of natural, plant-based antihistamines.

## Ethical approval:

The authors declare that due to an *in silico* analysis this study does not need any ethical approval.

## Consent to participate:

Not applicable

## Consent to publish:

Not applicable

## Author contributions:

All authors have equally contributed in the manuscript preparation like concept, designing, and collection of the data, as well as the writing and revision of the article.

## Funding:

The authors declare that no funds, grants, or other support were received during the preparation of this manuscript.

## Availability of data and materials:

All the data's are available with authors upon reasonable request

## Source of funding:

No funds, grants, or other support was received for this work.
